# A case report of jejunogastric intussusception complicated by torsion: 17 years after subtotal gastrectomy

**DOI:** 10.3389/fsurg.2026.1812043

**Published:** 2026-06-24

**Authors:** Haoyu Zhu, Lei Peng, Haipeng Liu

**Affiliations:** 1 The Second Clinical Medical College, Lanzhou University, Lanzhou, China; 2 Department of General Surgery, Lanzhou University Second Hospital, Lanzhou, China

**Keywords:** billroth 2 anastomosis, case report, gastrectomy, jejunogastric intussusception, postoperative complication, torsion

## Abstract

Jejunogastric intussusception (JGI) with torsion is a rare but life-threatening late complication after gastrectomy. We report a 65-year-old man who presented with acute epigastric pain, vomiting, and hematemesis 17 years after subtotal gastrectomy with retrocolic Billroth 2 reconstruction. Abdominal CT showed JGI with a target-like lesion and a whirl sign near the gastrojejunostomy, suggesting associated torsion and threatened bowel viability. Emergency laparotomy revealed extensive adhesions, 360° clockwise torsion of the efferent loop, and efferent-loop intussusception with retrograde herniation into the remnant stomach, resulting in severe edema, incarceration, ischemia, and necrosis. After adhesiolysis, abdominal reduction was unsuccessful; therefore, gastrotomy-assisted reduction, resection of the necrotic bowel, and side-to-side jejunojejunostomy were performed. The patient recovered uneventfully and was discharged on postoperative day 10. At 6-month follow-up, he remained asymptomatic, and contrast-enhanced CT showed no recurrent torsion or intussusception. This case highlights that JGI with torsion should be considered in patients with a history of gastrectomy who develop acute abdominal pain, vomiting, or hematemesis, even many years after surgery. Prompt CT-based diagnosis and definitive surgical treatment are essential when ischemia is suspected.

## Introduction

Jejunogastric intussusception (JGI) is an uncommon but severe postgastrectomy complication, which can occur anywhere from several days to 55 years postoperatively. Its incidence among patients with a history of gastrectomy is less than 0.1% (1–3). For patients presenting with hematemesis, abdominal pain, and a prior history of gastrectomy, JGI must be considered a potential etiology. Early diagnosis and prompt intervention are crucial to prevent the progression of complications, as timely surgical management relies heavily on early recognition. Emergency surgery remains the first-line treatment for acute JGI, with traditional laparotomy (including intestinal reduction or resection of the intussuscepted segment) as the primary surgical approach (3). Herein, we report an elderly patient who developed JGI with torsion 17 years after subtotal gastrectomy.

## Case presentation

A 65-year-old man was admitted to our hospital with acute upper abdominal pain, nausea, vomiting, and hematemesis. He had undergone subtotal gastrectomy with retrocolic Billroth 2 reconstruction and truncal vagotomy for perforated duodenal peptic ulcer in 2007, 17 years before the current presentation. The operative record from the initial surgery indicated that the afferent loop was approximately 15 cm long and the efferent loop approximately 20 cm long.

The patient developed upper abdominal pain, discomfort, nausea, and vomiting one day before admission, and his symptoms worsened after meals. The vomitus was grossly bloody. He had no melena, jaundice, chills, high fever, chest tightness, shortness of breath, or dyspnea. He also reported radiating pain to the shoulders, back, and waist. He first sought care at a local hospital, where emergency gastroscopy was attempted but terminated because severe gastric cavity compression and an unclear visual field caused by incarcerated edematous bowel prevented adequate evaluation. Abdominal CT at the local hospital suggested an internal hernia at the gastrojejunostomy site with possible intestinal necrosis (Figure 1), prompting urgent transfer to our tertiary care center.

**Figure 1:**
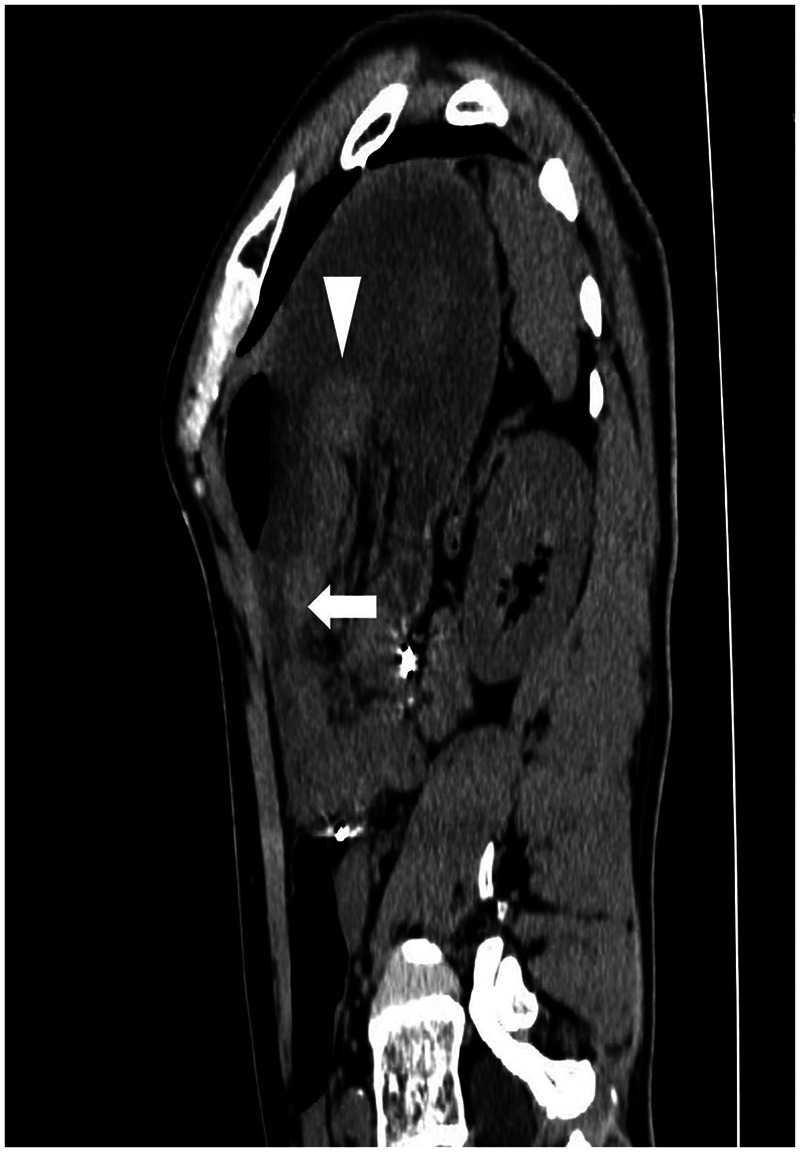
Abdominal CT scan in the oblique sagittal plane. The arrowhead indicates an edematous intestinal loop persistently incarcerated in the gastric cavity. The arrow indicates incarcerated mesentery and accompanying vessels with obvious compression, suggesting intestinal ischemia or necrosis.

After emergency surgical consultation, the preliminary diagnosis was acute ischemic bowel injury. Abdominal CT at our hospital showed JGI complicated by torsion after partial gastrectomy (Figure 2). Multiple small hepatic cysts, gallbladder stones, and bilateral renal calyceal calcifications were also noted. Indirect CT signs of torsion were present: the efferent loop showed a whirl sign near the gastrojejunostomy, and the mesenteric vessels had a spiral distribution, suggesting intestinal torsion with mesenteric twisting.

**Figure 2:**
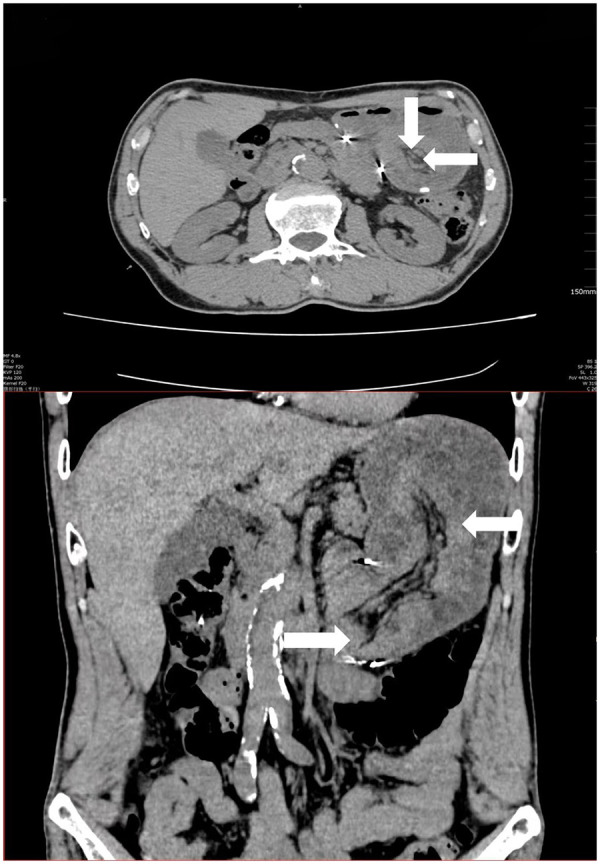
Abdominal CT scan. Upper image, axial view showing a typical target sign of intussusception; arrows indicate the core of the target sign. Lower image, coronal view showing jejunogastric intussusception; arrows indicate the jejunal loop invaginated into the remnant stomach.

Based on the patient’s history, symptoms, imaging findings, and concern for ischemia, emergency laparotomy was performed. On entering the abdominal cavity, extensive adhesions were identified among the upper abdominal intestines, abdominal wall, and liver. During adhesiolysis, the efferent loop was found to have a 360° clockwise torsion approximately 10 cm from the gastrojejunostomy. The mesentery of the twisted segment was tense and mildly congested. The torsion was closely associated with the intussuscepted segment and appeared to aggravate incarceration and ischemia.

After careful adhesiolysis, the stomach and transverse colon were exposed. The gastrojejunostomy was confirmed to be a retrocolic Billroth 2 anastomosis. The afferent loop appeared normal. Within the efferent loop, however, an intestinal segment approximately 15 cm distal to the gastrojejunostomy had intussuscepted into the proximal bowel (Figure 3). This efferent-loop intussusception, which subsequently prolapsed retrogradely through the gastrojejunostomy into the remnant stomach, constituted jejunogastric intussusception.

**Figure 3:**
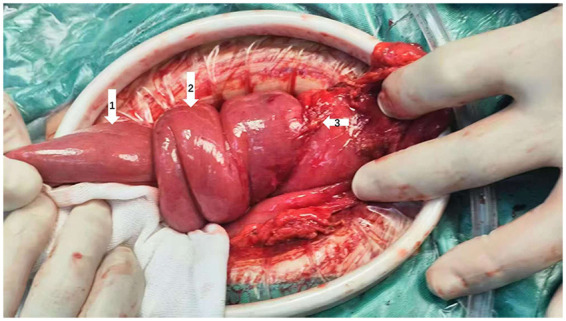
Intraoperative view showing the distal part of the efferent loop invaginating into the gastric remnant and forming jejunogastric intussusception. 1, efferent loop; 2, afferent loop; 3, gastrojejunostomy.

After the surrounding tissues were protected with gauze, a longitudinal incision of approximately 5 cm was made in the anterior gastric wall at the site closest to the incarcerated bowel. Approximately 600 mL of foul-smelling bloody fluid was evacuated. The intussuscepted bowel appeared dark red, thickened, edematous, avascular, and aperistaltic, consistent with intestinal necrosis (Figure 4), and it was resected. The remaining intussusception was gently reduced by traction along the intestinal axis using atraumatic intestinal forceps. Because 5 cm of both the proximal and distal jejunal stumps also appeared necrotic, these segments were resected with a 60-mm endoscopic linear cutter using a blue stapler cartridge.

**Figure 4:**
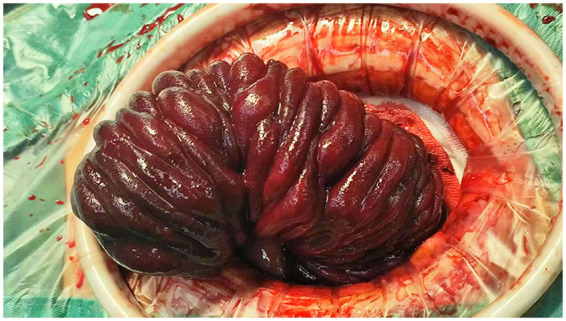
The intussuscepted bowel within the stomach appeared dark red, thickened, edematous, and aperistaltic, indicating intestinal necrosis.

The remaining bowel ends had good blood supply and normal peristalsis. A side-to-side jejunojejunostomy was performed using a 60-mm endoscopic linear cutter. The anastomosis was located 2 cm from the proximal stump and 8 cm from the distal stump, and the anastomotic opening was approximately 4 cm long. The common opening was closed with continuous 3–0 barbed sutures, and the seromuscular layer was reinforced with interrupted 3–0 Vicryl absorbable sutures. The anastomosis was patent, well vascularized, and leak-free. The small intestine was carefully returned to the abdominal cavity to avoid recurrent torsion. The gastric incision was closed with a linear cutter and reinforced with seromuscular sutures. A three-lumen gastrointestinal tube was placed 40 cm distal to the anastomosis under direct vision with guidewire assistance and fixed to the abdominal wall to ensure effective gastrointestinal decompression.

The abdominal cavity was irrigated with warm normal saline until the effluent was clear. Two abdominal drainage tubes and one pelvic drainage tube were inserted. The operation was completed without intraoperative complications, and estimated blood loss was 50 mL. Postoperatively, the patient was transferred to the intensive care unit for close monitoring and received antibiotic therapy, spasmolysis, analgesia, acid suppression, fluid resuscitation, and nutritional support. He recovered well, with no recurrent abdominal pain, vomiting, or hematemesis. He was discharged on postoperative day 10 after removal of the three-lumen gastrointestinal tube and resumption of a liquid diet. At the 1-week post-discharge follow-up, he tolerated a semi-liquid diet, had no abnormal abdominal signs, and abdominal ultrasound showed no intestinal obstruction or anastomotic effusion. At 6 months after surgery, he had resumed a regular diet and remained asymptomatic. Contrast-enhanced CT showed a patent anastomosis with no evidence of recurrent torsion or intussusception. The clinical timeline is summarized in Table 1.

**Table 1:** Clinical timeline.

Time point	Clinical event	Management and outcome
2007	Previous operation	Subtotal gastrectomy with retrocolic Billroth 2 reconstruction and truncal vagotomy for perforated duodenal peptic ulcer; the patient remained asymptomatic for 17 years.
1 day before admission	Upper abdominal pain, nausea, vomiting, and hematemesis developed and worsened after meals.	The patient sought emergency medical care.
Local hospital evaluation	Emergency gastroscopy was unsuccessful because incarcerated edematous bowel compressed the gastric cavity; CT suggested internal hernia and possible intestinal necrosis.	Urgent transfer to a tertiary care center was arranged.
Admission to tertiary hospital	CT showed jejunogastric intussusception with a target sign and a whirl sign near the gastrojejunostomy, suggesting torsion and threatened bowel viability.	Acute ischemic bowel injury was suspected, and emergency laparotomy was indicated.
Emergency surgery	Laparotomy revealed extensive adhesions, 360° clockwise efferent-loop torsion, and efferent-loop intussusception with retrograde herniation into the stomach; the bowel was edematous, incarcerated, ischemic, and necrotic.	Adhesiolysis, gastrotomy-assisted reduction, necrotic bowel resection, and side-to-side jejunojejunostomy were performed.
Postoperative days 1–9	No recurrent abdominal pain, vomiting, or hematemesis occurred.	The patient received intensive monitoring, gastrointestinal decompression, antibiotic therapy, acid suppression, fluid resuscitation, analgesia, and nutritional support.
Postoperative day 10	The patient was clinically stable and resumed oral liquid intake.	The gastrointestinal tube was removed, and the patient was discharged.
1 week after discharge	The patient tolerated a semi-liquid diet and had no abnormal abdominal signs.	Ultrasound showed no intestinal obstruction or anastomotic effusion.
6 months after surgery	The patient had resumed a regular diet and remained asymptomatic.	Contrast-enhanced CT showed a patent anastomosis and no recurrent torsion or intussusception.

## Patient perspective

From the patient’s perspective, the symptoms developed suddenly and progressed rapidly, especially the severe epigastric pain, repeated vomiting, and hematemesis, which caused marked anxiety and prompted him to seek emergency medical attention. He reported substantial relief after timely surgical treatment and was satisfied with the postoperative recovery and gradual return to oral intake. He also agreed to share his experience in the hope that this case may improve awareness of this rare postgastrectomy complication.

## Discussion

Jejunogastric intussusception (JGI) is a rare but clinically important late complication after gastrectomy. Although adult intussusception accounts for only 5%–10% of all intussusception cases (4), JGI may progress rapidly to obstruction, strangulation, and bowel necrosis. The clinical value of the present case lies in its unusual mechanism and delayed presentation rather than in the operative details. Two features make this case distinctive: the very long interval of 17 years after subtotal gastrectomy and the coexistence of efferent-loop JGI with 360° torsion near the gastrojejunostomy.

The mechanism in this patient was probably multifactorial. Retrocolic Billroth 2 reconstruction may create a confined retromesocolic route and alter the mobility of the afferent and efferent loops. Over time, postoperative adhesions can further fix the bowel and mesentery, predisposing the efferent loop to torsion and preventing spontaneous reduction once intussusception occurs. Truncal vagotomy may also contribute by impairing gastrointestinal motility and promoting abnormal peristaltic coordination. These anatomical and functional factors may explain why torsion and intussusception reinforced each other in this case, resulting in rapid vascular compromise and necrosis.

From a diagnostic perspective, this case emphasizes that JGI should remain in the differential diagnosis for any patient with a history of gastrectomy who presents with acute epigastric pain, vomiting, hematemesis, or signs of bowel obstruction, even many years after surgery. According to Shackman’s classification, this case corresponds to Type II JGI involving the efferent loop (5), the most frequently reported subtype (6). However, the combined findings of a target-like lesion within the stomach and a whirl sign at the anastomotic region are particularly important because they suggest not only intussusception but also associated torsion and threatened bowel viability. Early CT assessment is therefore central to timely surgical decision-making (7).

The therapeutic implication is that management should be guided by bowel viability and recurrence risk rather than by reduction alone. Acute JGI can deteriorate quickly, and delayed intervention has been associated with markedly increased mortality (5, 8). Although manual reduction may be feasible in selected patients with viable bowel, reduction alone has been linked to recurrence and may be unsafe when the bowel is edematous, ischemic, or necrotic (9). In the present case, emergency laparotomy with resection of nonviable bowel and reconstruction of intestinal continuity was clinically justified and resulted in an uneventful recovery without recurrence at the 6-month follow-up. This case therefore supports early recognition, prompt CT-based evaluation, and definitive surgical treatment when ischemia or torsion is suspected.

## Conclusion

JGI complicated by torsion is a rare but life-threatening late complication after gastrectomy. In patients with a history of gastrectomy who develop acute epigastric pain, vomiting, hematemesis, or signs of bowel obstruction, JGI should remain an important differential diagnosis even many years after the original operation. CT findings such as an intragastric target sign and a whirl sign near the gastrojejunostomy can help identify intussusception with associated torsion and threatened bowel viability. Prompt surgical intervention, with treatment tailored to bowel viability, is essential to prevent strangulation, necrosis, and recurrence.

## Data Availability

The original contributions presented in the study are included in the article/Supplementary Material, further inquiries can be directed to the corresponding author.
